# Association of Lung CT Findings in Coronavirus Disease 2019 (COVID-19) With Patients' Age, Body Weight, Vital Signs, and Medical Regimen

**DOI:** 10.3389/fmed.2022.912752

**Published:** 2022-06-30

**Authors:** Abdel-Ellah Al-Shudifat, Ali Al-Radaideh, Shatha Hammad, Nawal Hijjawi, Shaden Abu-Baker, Mohammed Azab, Reema Tayyem

**Affiliations:** ^1^Faculty of Medicine, The Hashemite University, Zarqa, Jordan; ^2^Prince Hamza Hospital, Amman, Jordan; ^3^Department of Medical Imaging, Faculty of Applied Medical Sciences, The Hashemite University, Zarqa, Jordan; ^4^Department of Nutrition and Food Technology, Faculty of Agriculture, The University of Jordan, Amman, Jordan; ^5^Department of Medical Laboratory Sciences, Faculty of Applied Medical Sciences, The Hashemite University, Zarqa, Jordan; ^6^Department of Pathology, Faculty of Medicine, Jordan University of Science and Technology, Irbid, Jordan; ^7^Department of Human Nutrition, and Biomedical and Pharmaceutical Research Unit, College of Health Sciences, QU Health, Qatar University, Doha, Qatar

**Keywords:** lung CT findings, COVID-19, risk factors, body weight, vital signs, medical regimen

## Abstract

**Objective:**

This study aimed to detect possible associations between lung computed tomography (CT) findings in COVID-19 and patients' age, body weight, vital signs, and medical regimen in Jordan.

**Methods:**

The present cross-sectional study enrolled 230 patients who tested positive for COVID-19 in Prince Hamza Hospital in Jordan. Demographic data, as well as major lung CT scan findings, were obtained from the hospital records of the COVID-19 patients.

**Results:**

The main observed major lung changes among the enrolled COVID-19 patients included ground-glass opacification in 47 (20.4%) patients and consolidation in 22 (9.6%) patients. A higher percentage of patients with major lung changes (24%) was observed among patients above 60 years old, while (50%) of patients with no changes in their lung findings were in the age group of 18–29 years old. Results obtained from the present study showed that only patients with major CT lung changes (9.7%) were prescribed more than three antibiotics. Additionally, 41.6 % of patients with major lung CT scan changes had either dry (31.0%) or productive (10.6%) cough at admission.

**Conclusion:**

Several factors have been identified by this study for their ability to predict lung changes. Early assessment of these predictors could help provide a prompt intervention that may enhance health outcomes and reduce the risk for further lung changes.

## Introduction

An outbreak of coronavirus disease-19 (COVID-19) infection began in December 2019 in Wuhan, the capital of central China's Hubei province (1). The COVID-19 pandemic has changed the focus of healthcare practitioners around the globe to control the spread of this contagious virus. In December 2020, there have been 73,275,943 confirmed cases of COVID-19 worldwide, including 1,650,348 confirmed deaths, which have been reported to the world health organization (WHO). Jordan is part of the worldwide pandemic of COVID-19 (2), where the virus started to spread rapidly among the population over the past few months, with currently 271,215 confirmed cases and 3,518 confirmed deaths according to the Ministry of Health daily reports (3).

COVID-19 can affect anyone causing mild to severe symptoms depending on the age of the infected person and the presence of certain underlying medical conditions. These medical conditions include cancer, smoking, obesity (body mass index (BMI) of 30 kg/m^2^ or higher), heart diseases, chronic obstructive pulmonary disease, chronic kidney diseases, immunocompromised state, pregnancy, type 2 diabetes, and sickle cell anemia (4). Moreover, it was found that a higher risk of COVID-19 infection was associated with male gender, Asian race, black/African American race, low air quality, housing instability, being unable to regularly move from place to place in a safe and timely manner, and living in senior living communities (5).

The clinical manifestations of COVID-19 include fever, cough, myalgia or fatigue, dyspnea, shortness of breath, and/or sore throat. More severe symptoms have been reported in patients with the aforementioned underlying medical conditions and those patients require hospitalization, admission to the Intensive Care Unit (ICU), intubation, or mechanical ventilation. Death was also reported to be very common among these critically ill patients (6, 7). The severity of these clinical signs experienced by COVID-19 patients was found to be highly associated with the degree of lung abnormality (8).

Radiological examinations are of paramount value in the detection, management, and follow-up of COVID-19. High-resolution, spiral chest CT examination is considered to be the first-line imaging modality in the diagnosis of COVID-19 as the lung was found to be the most affected organ among COVID-19 patients. The most common CT findings in COVID-19 patients include ground-glass opacification (GGO), calcified nodules, atelectasis, pleural effusion, infiltration, consolidation, fibrotic band, pneumonia, and emphysematous changes (9).

Few studies are currently available regarding the association of lung CT findings in COVID-19 with patients' medical prognosis in the Middle East (10–12). Thus, the main aim of the current study was to detect any possible associations between lung CT findings/changes in COVID-19 with patients' age, body weight, vital signs, and medical regimen in Jordan as one of the Middle East countries.

## Methods

### Data Collection

A cross-sectional, hospital-based study (*N* = 230, mean age 42.3, SD 15.4, males/females = 160/70) was conducted between March 17th and September 7th, 2020 to investigate the association between CT features, treatment course, and vital signs. The study was carried out at Prince Hamza Hospital; the main hospital that was allocated for COVID-19 patients in Jordan who were confirmed to be infected with the virus upon testing nasopharyngeal swabs using polymerase chain reaction (PCR).

All patients underwent unenhanced chest CT examinations covering from the apex to the lung base, in the supine position, using Philips MX-16 slices scanner (Philips Medical Solutions, The Netherlands). The scanning parameters were as follows: reconstruction matrix of 512 ×512 mm, voxel size of 1 x 1 x 2 mm (3), tube voltage, 140 kV; tube current, 217 mA. CT images of each patient at admission were assessed for the presence and distribution of parenchymal abnormalities, including ground-glass opacification (GGO), consolidation, multifocality, distribution (peripheral or diffuse), septal thickening, crazy paving, pulmonary nodules, and pleural effusion. Major lung CT scan changes were considered if bilateral involvement of more than two lobes of the peripheral lung with parenchymal abnormalities such as GGO mixed with multifocal consolidative opacities, septal thickening (with or without the nodular pattern), and the development of a crazy-paving pattern. Otherwise, parenchymal abnormalities were considered minor.

Data regarding patient's age (years), anthropometric [height (cm), weight (kg), BMI (kg/m^2^)], vital signs [pulse rate (bpm), respiratory rate (bpm), the temperature at admission, the temperature at discharge, systolic blood pressure (mmHg), diastolic blood pressure (mmHg), O_2_ saturation on room air (%), and O_2_ saturation at discharge (%)] were collected from the patients' medical files. However, some missing data regarding age, body weight and height were collected by phone directly from the patients. Furthermore, data about medications and supplements (antimalarial, number of antibiotics, respiratory antihistamines, steroids, ascorbic acid, vitamin B-complex supplement, zinc sulfate supplement), the severity of symptoms, oxygen therapy, cough at admission, cough at discharge, and daily number of smoked cigarettes were all collected from the patients' medical files. According to the patients' symptoms, laboratory results and imaging findings at admission, the disease severity of COVID-19 patients can be divided into three types: mild, moderate, and severe (13). The mild type was defined as having no symptoms or complaining of one or two symptoms that last for a few days. The moderate type was defined as having more complicated symptoms that last for a longer duration and/or having major lung CT scan findings. The severe type was defined as having symptoms similar to those of the moderate type in addition to the need for oxygen therapy (13).

The present study was approved by the Ethics Committee of Prince Hamza Hospital (1,630/1) and all patients gave verbal consent to participate at the start of the phone call.

### Statistical Analysis

Analyses were performed in SPSS version 26. Data are presented as mean ± standard deviation (SD) with percentages for descriptive statistics. One-Way ANOVA with LSD (Least Significant Difference) *Post Hoc* Test was used to find the differences among the three levels of lung CT scan changes (normal, minor abnormalities, and major abnormalities). A chi-square (χ (2)) was used to find differences between categorical variables. Linear regression was used to find the association between lung CT scan findings and some variables. *p-*value was set at ≤ 0.05.

## Results

**Figure 1:**
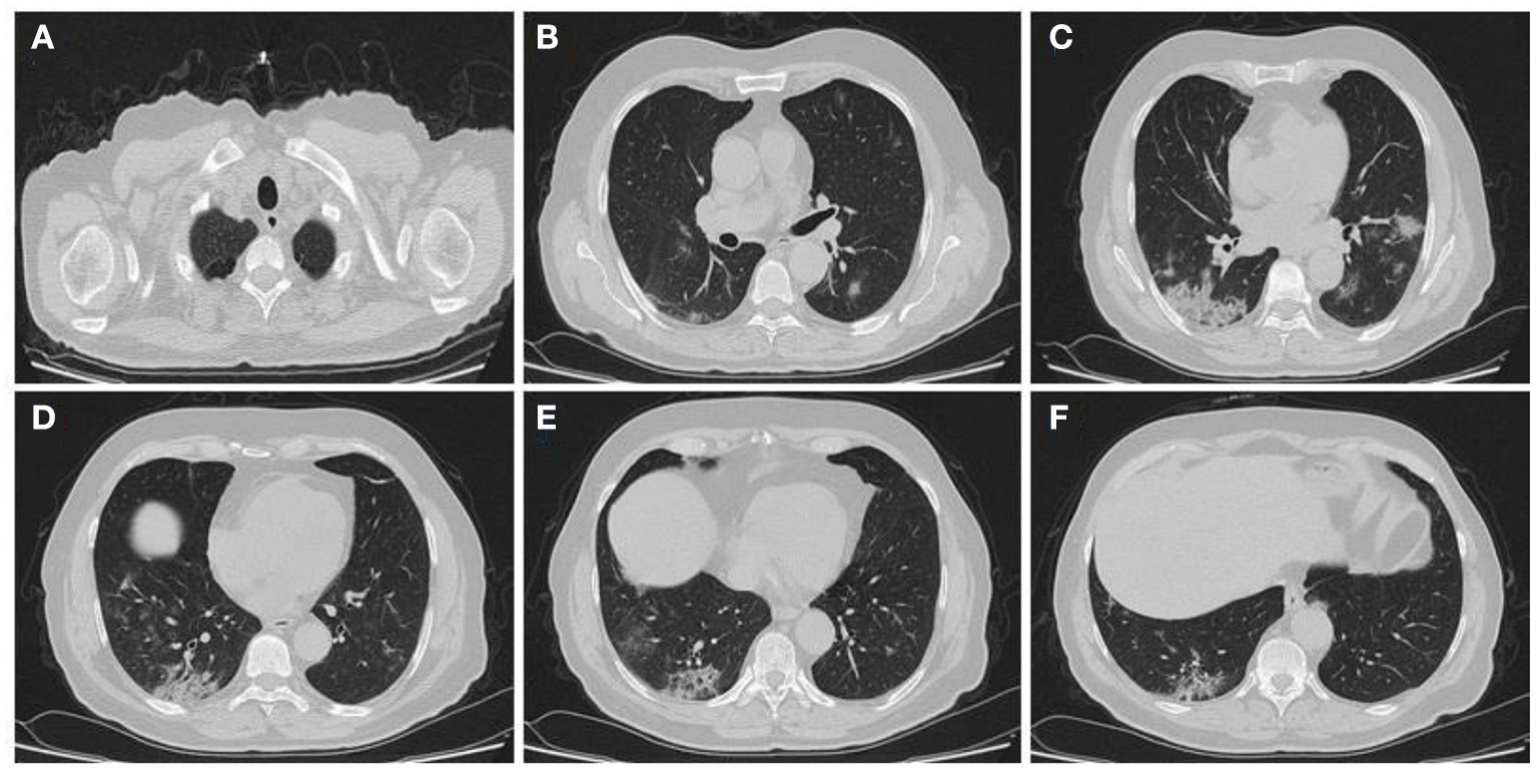
High resolution, axial non-enhanced spiral chest CT images (lung window) of a 69-years old patient who was confirmed to be infected by COVID-19 and admitted to hospital with fever and dry cough. CT images show **(A)** emphysematous changes in the apices of both lungs and multiple patchy consolidations predominantly in the lower lobe of the right lung **(B–F)**.

**Figure 2:**
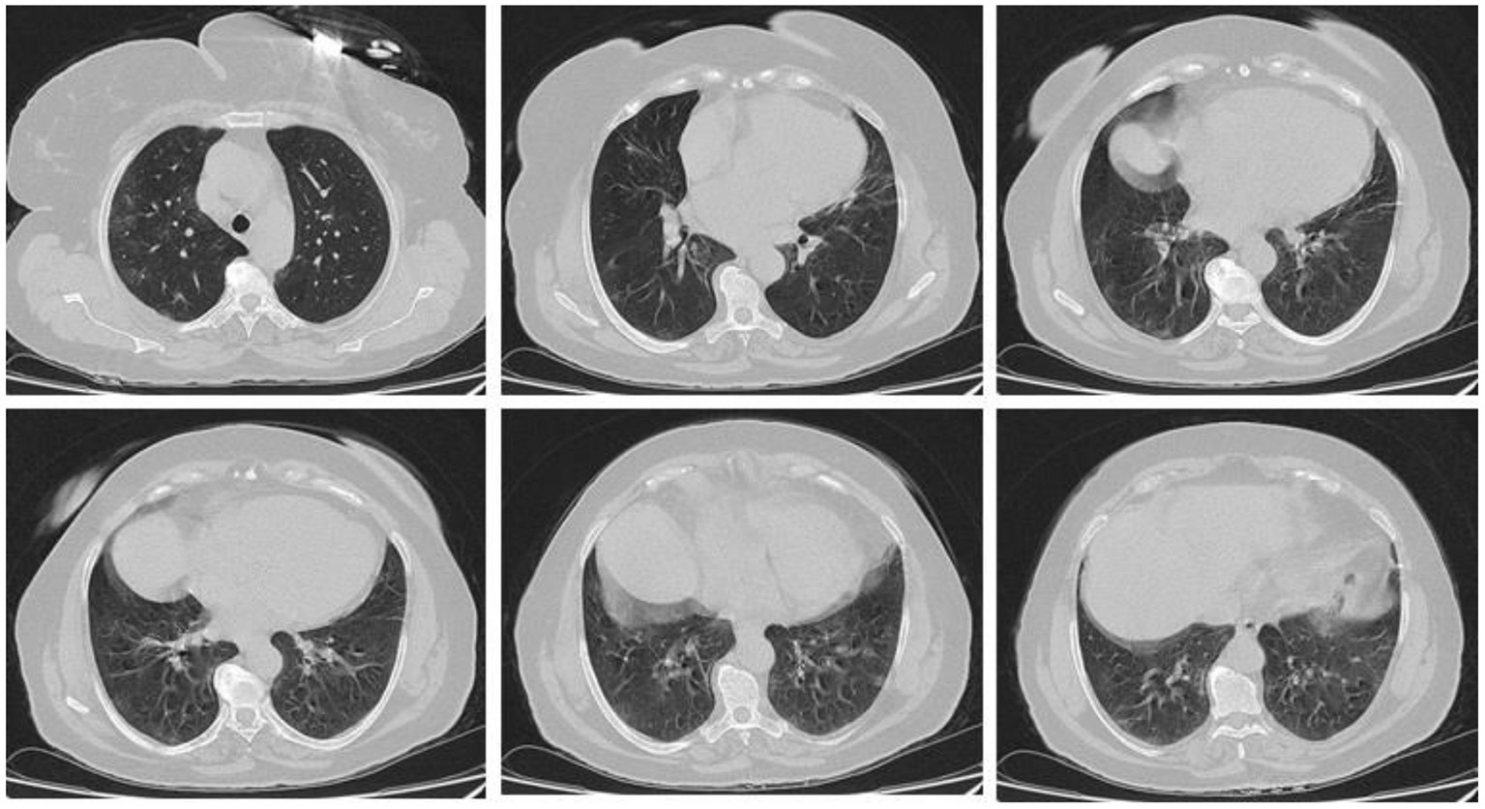
High resolution, axial non-enhanced spiral chest CT images (lung window) of a 59-years old patient who was confirmed to be infected by COVID-19 and admitted to hospital with Flu-like symptoms and gastrointestinal tract symptoms (mostly diarrhea). CT images show multiple faint patchy consolidations disseminated in ground-glass patterns.

**Figure 3:**
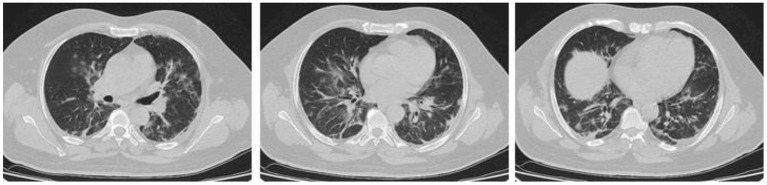
High resolution, axial non-enhanced spiral chest CT images (lung window) of a 50-years old patient who was confirmed to be infected by COVID-19 and admitted to hospital with fever and dry cough. CT images show several airspace opacities, ground-glass shadows, and multiple sub-segmental consolidations most pronounced in the lower lobe of both lungs.

**Figure 4:**
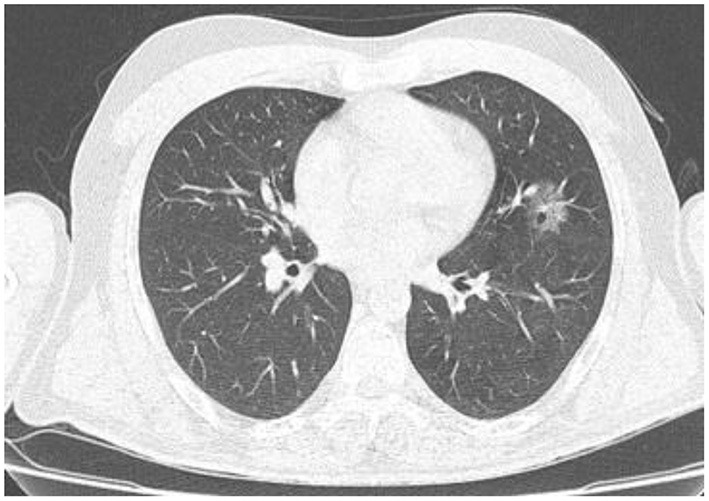
High resolution, axial non-enhanced spiral chest CT image (lung window) of a 44-years old patient who was confirmed to be infected by COVID-19 and admitted to hospital with no symptoms (asymptomatic). CT image shows only a small ground-glass pattern in the lower aspect of the anterior segment of the upper lobe of the left lung.

In the present study, 160 males and 70 females were enrolled to explore the association of lung CT findings in COVID-19 with patients' age, body weight, vital signs, and medical regimen. The reported major lung CT scan findings were GGO in 47 (20.4%) patients; calcified nodules in 3 (1.3%) patients; atelectasis and fibrotic band in 16 (7.0%) patients; pleural effusion and consolidation in 22 (9.6%) patients; infiltration and pneumonia in 11 (4.8%) patients; and emphysematous changes in 11 (4.8%) patients of the study sample (230 participants). Three different cases with major lung changes are shown in Figures 1–3. However, one case with minor lung CT scan findings is shown in Figure 4.

The main characteristics of the study participants shown in Table 1 were categorized based on the three grades of lung CT findings. A significant difference (*p* ≤ 0.05) was detected among the different age groups for each lung CT scan findings. Young age (18–29 years) seems to be protected against minor and major lung changes while they accounted for the highest percentage of patients (50%) with no changes in their lungs. However, the highest percentage of major lung changes was 24.1% which was observed in patients above 60 years old. Regarding BMI, a higher percentage of obesity was observed in patients with both minor and major lung CT scan findings (35.7 and 34.7%, respectively). From the study population, 161 (70.3%), 55 (24.0%), and 10 (4.4%) patients were classified as mild, moderate, and severe, respectively. However, COVID-19 consequences were fatal in 3 (1.3%) patients (with a median age of 80 years).

**Table 1:** Frequencies and percentages of sex, age, and medical variables among the study participants based on lung CT abnormalities.

**Variables**	**Lung CT abnormalitiesn (%)**				***p–*value**
	**Normal N = 78**	**Minor abnormality N = 37**	**Major abnormality N = 115**	**Total *N* = 230**	
**Sex**					0.908
Male	50 (64.1)	26 (70.3)	84 (73.0)	160 (69.5)	
Female	28 (35.9)	11 (29.7)	31 (27.0)	70 (30.5)	
**Age group**					0.001
18–29	39 (50.0)	5 (13.9)	8 (6.9)	52 (22.6)	
30–39	22 (28.2)	6 (16.7)	12 (10.3)	40 (17.4)	
40–49	10 (12.8)	13 (36.1)	31 (26.7)	54 (23.5)	
50–59	5 (6.4)	5 (13.9)	37 (31.9)	47 (20.4)	
60–69	1 (1.3)	5 (13.9)	18 (15.5)	24 (10.4)	
70–79	1 (1.3)	2 (5.6)	7 (6.0)	10 (4.3)	
>80	0 (0)	0 (0)	3 (2.6)	3 (1.3)	
**BMI**					0.034
<18.5	1 (1.4)	0 (0.0)	0 (0.0)	1 (0.5)	
18.5–24.9	29 (41.4)	10 (35.7)	20 (20.4)	59 (30.1)	
25–29.9	23 (32.9)	8 (28.6)	44 (44.9)	75 (38.3)	
30–34.9	13 (18.6)	9 (32.1)	20 (20.4)	42 (21.4)	
>35	4 (5.7)	1 (3.6)	14 (14.3)	19 (9.7)	
**Severity of symptoms**					0.174
Mild	67 (87.0)	29 (78.4)	65 (56.5)	161 (70.3)	
Moderate	10 (13.0)	8 (21.6)	37 (32.2)	55 (24.0)	
Severe	0 (0)	0 (0)	10 (8.7)	10 (4.4)	
Fatal	0 (0)	0 (0)	3 (2.6)	3 (1.3)	
**Vitamin C supplement**					0.251
Yes	43 (55.1)	20 (54.1)	71 (61.7)	134 (58.5)	
No	35 (44.9)	17 (45.9)	43 (37.4)	95 (41.5)	
**Vitamin B-complex supplement**					0.473
Yes	14 (17.9)	14 (37.8)	43 (37.4)	71 (30.9)	
No	64 (82.1)	23 (62.2)	72 (62.6)	159 (69.1)	
**Zinc sulfate supplement**					
Yes	31 (39.7)	19 (51.4)	57 (49.6)	107 (46.5)	0.324
No	47 (60.3)	18 (48.6)	58 (50.4)	123 (53.5)	
**Antimalarial medication**					0.119
Prescribed	47 (60.3)	17 (45.9)	56 (48.7)	120 (52.2)	
Not Prescribed	30 (38.5)	19 (51.4)	57 (49.6)	106 (46.1)	
Hold or give with attention	1 (1.3)	1 (2.7)	2 (1.7)	4 (1.7)	
**Number of antibiotics**					0.020
None	70 (89.7)	34 (91.9)	61 (53.5)	165 (72.1)	
One	8 (10.3)	1 (2.7)	26 (22.8)	35 (15.2)	
Two	0 (0.0)	2 (5.4)	16 (14.0)	18 (7.9)	
Three	0 (0.0)	0 (0.0)	10 (8.8)	10 (4.4)	
Four	0 (0.0)	0 (0.0)	1 (0.9)	1 (0.4)	
**Steroids**					0.143
Prescribed	2 (2.6)	3 (8.1)	25 (21.7)	30 (13.0)	
Not Prescribed	76 (97.4)	34 (91.9)	90 (78.3)	200 (87.0)	
**Respiratory antihistamines medications**					0.072
Prescribed	7 (9.0)	5 (13.5)	34 (29.6)	46 (20.0)	
Not prescribed	71 (91.0)	32 (86.5)	81 (70.4)	184 (80.0)	
**Oxygen therapy**					0.116
Not Used	76 (97.4)	37 (100)	95 (82.6)	208 (90.4)	
O2 mask cannula	2 (2.6)	0 0	18 (15.7)	20 (8.7)	
Ventilator	0 (0)	0 (0)	2 (1.7)	2 (0.9)	
**Temperature at admission**					0.772
Normal	60 (76.9)	27 (73.0)	68 (60.2)	155 (68.0)	
Elevated	18 (23.1)	10 (27.0)	45 (39.8)	73 (32.0)	
**Temperature on discharge**					0.228
Normal	78 (100)	37 (100)	110 (97.3)	225 (98.7)	
Elevated	0 (0)	0 (0)	3 (2.7)	3 (1.3)	
**Cough at admission**					0.037
No cough	67 (85.9)	30 (81.1)	66 (58.4)	163 (71.5)	
Dry cough	8 (10.3)	6 (16.2)	35 (31.0)	49 (21.5)	
Productive cough	3 (3.8)	1 (2.7)	12 (10.6)	16 (7.0)	
**Cough on discharge**					0.133
No cough	78 (100)	37 (100)	108 (97.3)	223 (98.7)	
Dry cough	0 (0)	0 (0)	3 (2.7)	3 (1.3)	

*The p-value for chi-square ≤ 0.05 is considered significant*.

The results of the present study showed that more than two antibiotics were prescribed to 27 out of 115 patients with major CT lung findings. On the other hand, only two patients with normal and minor CT scan abnormalities were given two antibiotics to protect them from secondary bacterial infection. Additionally, 41.6 % of patients with major lung CT scan findings had either dry (31.0%) or productive (10.6%) cough at admission.

Table 2 presents the means of anthropometric and medical variables among the study participants based on lung CT scan abnormalities. Significantly (*p* ≤ 0.05) higher values of age [mean (SD): 50.8 (13.8) years), weight (85.4 (15.1) kg], and BMI [29.4(5.2) kg/m^2^] were observed among patients with major lung CT scan findings as compared with patients who had no or minor CT scan findings.

**Table 2:** Means of anthropometric and medical variables among the study participants based on lung CT abnormalities.

**Variable**	**Lung CT abnormalities**				***p-*value**
	**Normal**	**Minor abnormality**	**Major abnormality**	**Total**	
Age (years)	31.5 (11.5)^a^	45.2 (14.9)^b^	50.8 (13.8)^c^	43.4 (15.8)	0.001
Height (cm)	172.8 (10.5)	171.4 (10.5)	170.7 (9.2)	171.6 (9.8)	0.510
Weight (kg)	77.7 (18.1)^a^	77.8 (10.6)^a^	85.4 (15.1)^b^	81.6 (16.1)	0.020
BMI (kg/m^2^)	25.9 (5.1)^a^	26.8 (4.9)^a^	29.4 (5.2)^b^	27.7 (5.3)	0.001
Pulse rate (bpm)	81.22 (8.53)	84.21 (7.58)	81.4 (8.32)	81.77 (8.31)	0.185
Respiratory rate (bpm)	19.91 (8.60)	18.67 (1.79)	19.45 (5.21)	19.47 (6.19)	0.642
Systolic blood pressure (mmHg)	121.0 (9.8)	122.6 (8.8)	123.7 (13.6)	122.7 (11.8)	0.331
Diastolic blood pressure (mmHg)	74.9 (7.6)	75.9 (7.3)	77.5 (10.3)	76.4 (9.1)	0.191
O2 saturation on room air (%)	96.3 (4.6)	95.8 (3.2)	95.1 (4.2)	95.6 (4.2)	0.157
O2 saturation at discharge (%)	96.9 (2.5)	96.9 (2.1)	96.4 (3.1)	96.6 (2.7)	0.398
Number of cigarettes	8.1 (13.3)	2.5 (6.8)	4.9 (11.0)	5.9 (11.7)	0.153

*Data are presented as mean ± SD Significance was set at p ≤ 0.05; ANOVA and LSD tests were used to assess the significance. Different three letters (a,b,c) mean that there is a significant difference between three variables and the same letters mean that there are no significant differences between variables*.

The associations between lung CT scan changes and the possible predictors are shown in Table 3. While significant direct associations were observed between lung CT scan changes and age, BMI, number of antibiotics, steroids, the severity of symptoms, and cough at admission, a marginally significant negative association was detected with O_2_ saturation on room air at admission.

**Table 3:** Predictors of lung CT changes among the study participants.

**Variable**	**R^**2**^**	**ANOVA**	**Model**	**B**	**ß**	***p-*value**
Lung CT scan changes	0.295	F=95.239	Age	0.031	0.543	0.001
	0.000	F=0.005	Sex	0.009	0.005	0.945
	0.096	F=14.413	BMI	0.054	0.310	0.001
	0.005	F=1.243	Antimalarial	−0.123	−0.074	0.266
	0.066	F = 16.089	Number of Antibiotics	0.252	0.257	0.001
	0.022	F = 5.190	Respiratory Antihistamines	0.337	0.149	0.024
	0.017	F = 3.872	Steroids	0.346	0.129	0.050
	0.002	F = 0.405	Ascorbic Acid	0.071	0.042	0.525
	0.006	F = 1.419	Vitamin B–Complex Supplement	0.149	0.079	0.235
	0.005	F = 1.058	Zinc Sulfate Supplement	0.124	0.068	0.305
	0.023	F = 5.306	Severity of Symptoms	0.231	0.151	0.022
	0.000	F = 0.001	Pulse Rate	0.000	−0.002	0.971
	0.001	F = 0.159	Respiratory Rate	−0.004	−0.028	0.690
	0.010	F = 2.217	Systolic Blood Pressure	0.008	0.101	0.138
	0.015	F = 3.321	Diastolic Blood Pressure	0.012	0.123	0.070
	0.017	F = 3.722	O2 Saturation on room air at admission	−0.028	−0.130	0.051
	0.008	F = 3.163	O2 Saturation at discharge	−0.028	−0.087	0.201
	0.016	F = 3.613	Oxygen Therapy	0.350	0.125	0.059
	0.002	F = 0.502	Temperature at Admission	−0.091	−0.047	0.479
	0.011	F = 2.578	Temperature on Discharge	0.841	0.106	0.110
	0.038	F = 8.861	Cough at Admission	0.284	0.194	0.003
	0.015	F = 3.524	Cough During Discharge	0.852	0.124	0.062

*p ≤ 0.05. Linear regression was used to assess predictors of lung CT changes among the study participants*.

## Discussion

The severity of COVID-19 infection is multifactorial in nature and could be influenced by various medical and personal variables. Understanding the association between such factors and pathological lung changes would allow better utilization of the existing medical resources and alleviate the consequences of COVID-19 infection. A thorough recognition of the pathophysiological determinants of COVID-19 infection may promote a more complete comprehension of a patient's clinical presentation and management.

In agreement with our findings, previous studies showed that the most common hallmark in the COVID-19 virus-affected lungs is GGO followed by consolidation (14, 15). The adverse manifestations progress during the course of COVID-19 infection. Early-phase of the disease was found to be characterized by the detection of GGO (16). Also, the increased density of GGO lesions could be used as a marker for disease progression (15, 17). A study by Wang et al. (15) revealed an increase in GGO in the late stages of the disease (15).

Similar to our findings, symptoms, including fever, cough, respiratory distress (lung injury that allows fluid to leak into the lungs), or requirement for mechanical ventilation, were previously reported to have higher intensity in patients with major lung changes detected by the CT scan (18, 19). On the other hand, a higher density of GGO, unlike consolidation, was reported to be associated with asymptomatic cases of COVID-19 infection (20). Based on the number of confirmed cases and deaths, the mortality rate of COVID-19 (~2%) is lower than other viruses within the same family (such as SARS; 9.5% mortality rate) (21). Solomon et al. (2021) identified GGO and subpleural bands with concomitant pulmonary function abnormalities in CT scans of patients infected with COVID-19 and indicated several predictors of lung changes including the need for intensive care unit admission, mechanical ventilation, higher inflammatory markers, and longer hospital stay (22). Additionally, lung changes detected by CT scan was documented to estimate the probability of lung malignancy (23). Many factors could influence the disease process and the severity of illness such as BMI and age; however, the ability to identify such factors is limited due to the rapidly evolving pandemic.

CDC data indicated an increase in mortality rate from COVID-19 infection among people who are 60 years and older, mainly those who suffer from other underlying comorbidities (17). Here, we detected a direct positive trend (*p* = 0.001) between the increase in age and increased CT abnormalities, in which older participants had a spectrum of major CT scan findings. Although the severity of the disease augmented with age; however, the highest incidence was observed among younger participants. This finding is in agreement with the age group-specific pattern of the incidence in the United States and Europe, where the highest incidence was observed in the 20–29 years age group (24, 25). The elevated incidence of infection among young individuals can be justified by the fact that this group contributes to the vast majority of workers in industries and frontline occupations that are highly exposed to the transmission of the infection (24). Moreover, the unintentional spread of infection is high in the young age group due to the predominant lack of symptoms concomitant with the lack of proper social distancing (24, 25).

Expiratory reserve volume, functional capacity, and respiratory system compliance are negatively influenced by obesity, which suggests an aggressive course of COVID-19 infection (26, 27) and a risk of mortality in obese patients (28). The effects of obesity on respiratory functions could augment the increased need for careful attention and precise management to prevent the probable complications of the disease in this group of patients (26, 27).

## Study Limitation

Our study has several limitations worth noting, including the limited sample size; however, this study was performed at the early stages of the outbreak in Jordan where the number of confirmed infections was also limited. Also, a limited number of confounders was gathered from participants; however, the nature of the disease, the demand of limited exposure to the participant, and the lack of accompanying family members hindered the ability to collect a wide range of confounding variables.

## Conclusion

Many predictors of lung changes including age, BMI, number of antibiotics, steroids, the severity of symptoms, O_2_ saturation in room air and cough at admission as well as respiratory distress and requirement for ventilation had been identified among the study patients. Considering these factors could reduce the challenge confronted by physicians to provide effective treatment for patients infected with COVID-19. Performing an evaluation of the risk factors identified by this study for their association with lung changes, as detected by CT, would enhance providing early and optimal health care for infected patients even in the absence of instant accessibility of CT scans. As today's challenges are tomorrow's opportunities, emphasis on clinical and research activity would no doubt highly alleviate the impact of COVID on communities by utilizing research-based data for planning comprehensive mitigation plans for health care.

## Data Availability Statement

The raw data supporting the conclusions of this article will be available upon request from the corresponding author, without undue reservation.

## Ethics Statement

The study was approved by the IRB at Prince Hamza Hospital (1630/1), and all participants formally consented to participate in the study. The patients/participants provided their verbal informed consent to participate in this study.

## Author Contributions

RT, A-EA-S, and MA conceived designed and supervised the study. RT and SA-B were responsible for data entry, analysis, and interpretation. RT, NH, AA-R, SA-B, and SH drafted the manuscript. All authors critically reviewed the manuscript and approved the final version.

## Conflict of Interest

The authors declare that the research was conducted in the absence of any commercial or financial relationships that could be construed as a potential conflict of interest.

## Publisher's Note

All claims expressed in this article are solely those of the authors and do not necessarily represent those of their affiliated organizations, or those of the publisher, the editors and the reviewers. Any product that may be evaluated in this article, or claim that may be made by its manufacturer, is not guaranteed or endorsed by the publisher.
